# Supplementary material for: Theory of optical responses in clean multi-band superconductors
Source: Nat Commun. 2021 Mar 12;12:1617. doi: 10.1038/s41467-021-21905-x (PMC7954819; doi:10.1038/s41467-021-21905-x)
Supplement: Supplementary file 1 — Supplementary Information [file 41467_2021_21905_MOESM1_ESM.pdf]

Supplementary Information for "Theory of optical responses in clean multi-band superconductors"

Junyeong Ahn[1,2,*] and Naoto Nagaosa[2,3,†]

[1]*Department of Physics, Harvard University, Cambridge, Massachusetts 02138, USA*

[2]*RIKEN Center for Emergent Matter Science (CEMS), Wako, Saitama 351-0198, Japan*

[3]*Department of Applied Physics, The University of Tokyo, Bunkyo, Tokyo 113-8656, Japan*

(Dated: February 18, 2021)

Supplementary Note 1. Additional model calculations

We take two 2D models — respectively non-degenerate and twofold-degenerate at generic momenta — to further demonstrate our theory. The first is a model of single Dirac cone.

$$h_1 = -\mu + \hbar v k_x \sigma_y - \hbar v k_y \sigma_x, \tag{1}$$

where $\sigma_{i=x,y,z}$ is a spin Pauli matrix. It is symmetric under mirror $m_x = i\sigma_x$ and $m_y = i\sigma_y$, fourfold rotation $c_{4z} = e^{-i\pi\sigma_z/4}$, and time reversal $t = i\sigma_y K$. No mirror m_z and inversion p symmetries exist. Here, we use lowercase letters to denote symmetry operators of the normal state Hamiltonian to distinguish them from those of the BdG Hamiltonian. There are four possible superconducting pairing functions we list in Table. 1. When the pairing function is even (odd) under c_{2z}, the BdG Hamiltonian has symmetry under $\mathcal{C} = C_{2z}C$ where $\mathcal{C}^2 = -1$ $(+1)$ because $C_{2z} = c_{2z}$ $(C_{2z} = \tau_z c_{2z})$. In accordance with our general theory, conductivity tensors can be non-zero only when $\mathcal{C}^2 = 1$ if we keep $\mathcal{C}$ symmetry [Fig. 1(a)]. In the limit $\omega, \Delta \ll \mu$, the non-zero conductivity can be analytically calculated as $\sigma^{cc} \sim \frac{e^2}{h}\frac{1}{\hbar\omega}\frac{(\Delta_4 k_F)^2}{E_F}$, where $E_F = \mu$ and $k_F = \mu/\hbar v$ (see Supplementary Note 2). $\sigma^{xy} = \sigma^{yx} = 0$ is due to mirror symmetries in our model. Figure 1(b) shows the case where $\mathcal{C}$ symmetry is broken by a C_{2z}-parity mixing due to the additional s-wave pairing $\Delta_1 \neq 0$.

Next, we double the orbital degrees of freedom in Supplementary Eq. (1) and add a mass term to have a low-energy model of a doped 2D topological insulator.

$$h_2 = -\mu + \hbar v k_x \rho_x \sigma_y - \hbar v k_y \rho_x \sigma_x + M\rho_z, \tag{2}$$

where $\rho_{i=x,y,z}$ is an orbital Pauli matrix. It has $m_x = i\sigma_x$, $m_y = i\sigma_y$, $m_z = i\rho_z\sigma_z$, $c_{4z} = e^{-i\pi\sigma_z/4}$, and $t = i\sigma_y K$ symmetries. $pt = i\rho_z\sigma_y K$ symmetry imposes Kramers degeneracy at each $\mathbf{k}$, where $p = m_x m_y m_z$. Since this model is spin-orbit coupled, optical excitations can generally occur, as illustrated in Fig. 2(d) of the main text. When the pairing is odd-m_z, however, optical excitations are not allowed because of the combination of the selection rules by unitary and PC symmetries (see Methods section 7 in the main text). Figure 1(c,d) shows non-zero optical conductivity tensors for constant pairing functions in Table 2. Let us note that, while Δ_2 has the same symmetry properties as the s-wave pairing under mirror operations and opens the full gap, we have non-zero optical conductivity without breaking any symmetry. In the case of Δ_5 pairing, the response is

* junyeongahn@fas.harvard.edu
† nagaosa@riken.jp

<table>
<tr><th>$\Delta(\mathbf{k})$</th><th>m_x</th><th>m_y</th><th>c_{4z}</th><th>Node</th><th>Lowest excitation</th></tr>
<tr><td>$\Delta_1 i\sigma_y$</td><td>$+$</td><td>$+$</td><td>$+$</td><td>Gapped</td><td>No</td></tr>
<tr><td>$\Delta_2(k_x\sigma_x + k_y\sigma_y)i\sigma_y$</td><td>$-$</td><td>$-$</td><td>$+$</td><td>Gapped</td><td>No</td></tr>
<tr><td>$\Delta_3(k_x\sigma_x - k_y\sigma_y)i\sigma_y$</td><td>$-$</td><td>$-$</td><td>$-$</td><td>Point</td><td>No</td></tr>
<tr><td>$\Delta_{4a}k_x\sigma_z i\sigma_y$</td><td>$-$</td><td>$+$</td><td rowspan="2">$\begin{pmatrix} 0 & 1 \\ -1 & 0 \end{pmatrix}$</td><td>Line</td><td>Yes</td></tr>
<tr><td>$\Delta_{4b}k_y\sigma_z i\sigma_y$</td><td>$+$</td><td>$-$</td><td>Line</td><td>Yes</td></tr>
</table>

Supplementary Table 1: Time-reversal-symmetric pairing functions of a 2D single Dirac fermion model. Pairing functions are given up to the leading order in $\mathbf{k}$. The second column shows the matrix representation r_g defined by $u_g\Delta_i(\mathbf{k})u_g^T = (r_g)_{ij}\Delta_j(g\mathbf{k})$.

<table>
<tr><th>$\Delta(\mathbf{k})$</th><th>m_x</th><th>m_y</th><th>m_z</th><th>c_{4z}</th><th>Node</th><th>Lowest excitation</th></tr>
<tr><td>$\Delta_1 i\sigma_y$</td><td>$+$</td><td>$+$</td><td>$+$</td><td>$+$</td><td>Gapped</td><td>No</td></tr>
<tr><td>$\Delta_2\rho_z i\sigma_y$</td><td>$+$</td><td>$+$</td><td>$+$</td><td>$+$</td><td>Gapped</td><td>Yes</td></tr>
<tr><td>$\Delta_3\rho_x i\sigma_y$</td><td>$+$</td><td>$+$</td><td>$-$</td><td>$+$</td><td>Gapped</td><td>No</td></tr>
<tr><td>$\Delta_4\rho_y\sigma_z i\sigma_y$</td><td>$-$</td><td>$-$</td><td>$-$</td><td>$+$</td><td>Gapped</td><td>No</td></tr>
<tr><td>$\Delta_{5a}\rho_y\sigma_y i\sigma_y$</td><td>$-$</td><td>$+$</td><td>$+$</td><td rowspan="2">$\begin{pmatrix} 0 & -1 \\ 1 & 0 \end{pmatrix}$</td><td>Point</td><td>Yes</td></tr>
<tr><td>$\Delta_{5b}\rho_y\sigma_x i\sigma_y$</td><td>$+$</td><td>$-$</td><td>$+$</td><td>Point</td><td>Yes</td></tr>
</table>

Supplementary Table 2: Time-reversal-symmetric constant pairing functions of a 2D TI model. The second column shows the matrix representation r_g defined by $u_g\Delta_i(\mathbf{k})u_g^T = (r_g)_{ij}\Delta_j(g\mathbf{k})$.

highly anisotropic, and, in fact, $\sigma^{yy} = 0$ while $\sigma^{xx} \neq 0$. The vanishing of σ^{yy} is not due to symmetry. It is a coincidence due to the fact that $\partial_y h \propto \Delta$ (see Supplementary Note 3). We can generate $\sigma^{yy} \neq 0$, e.g., by adding quadratic terms in the mass term by $M \to M - k^2$. $\sigma^{xy} = \sigma^{yx} = 0$ is again due to mirror symmetries.

Supplementary Note 2. Analytic calculation of the optical conductivity in the superconducting state of a single Dirac model

Here we consider the Δ_{4a} pairing in Supplementary Eq. (1) such that the BdG Hamiltonian is

$$H = -\mu\tau_z + \hbar v k_x \tau_z \sigma_y - \hbar v k_y \sigma_x + \Delta_{4a} k_x \tau_x \sigma_x. \tag{3}$$

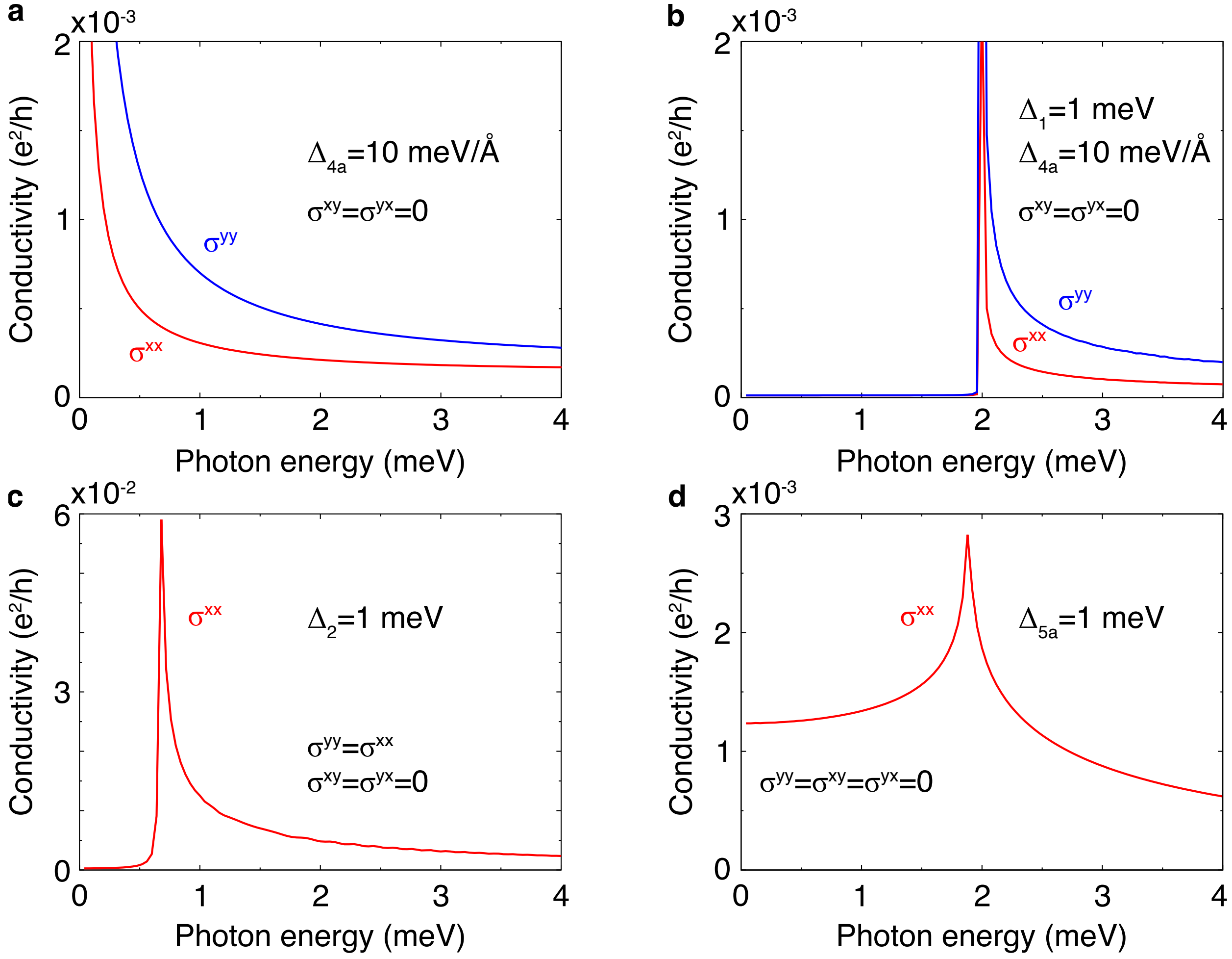

Supplementary Fig. 1: Intrinsic optical conductivity in 2D superconductor models. a,**b**, Superconducting state of Supplementary Eq.(1) with $\mu = 0.1$ eV, $\hbar v = 1$ eV/Å, and the order parameters listed in Table. 1. **c**,**d**, Superconducting state of Supplementary Eq.(2) with $\mu = 0.15$ eV, $\hbar v = 1$ eV/Å, $M = 0.05$ eV, and the order parameters listed in Table. 2. As the spectrum is gapless in **a** and **d**, optical conductivity is non-zero down to zero frequency.

It has $M_x = i\sigma_x$, $M_y = i\tau_z\sigma_y$, $C_{2z} = i\tau_z\sigma_z$, $T = i\sigma_y K$, and $C = \tau_x K$ symmetries. For a simpler understanding of the analytic calculation, we perform a unitary transformation $H \to U^{-1}HU$ using

$$U = \begin{pmatrix} 1 & 0 & 0 & 0 \\ 0 & 1 & 0 & 0 \\ 0 & 0 & 0 & 1 \\ 0 & 0 & 1 & 0 \end{pmatrix} = \frac{1}{2}\left(1 + \tau_z + \sigma_x - \tau_z\sigma_x\right) \tag{4}$$

such that the new Hamiltonian is

$$H = \hbar v(k_x\sigma_y - \hbar k_y\sigma_x) - \mu\tau_z + \Delta_{4a}k_x\tau_x. \tag{5}$$

In this form, since the σ and τ terms commute with each other, it is obvious that the energy eigenstate takes the form,

$$|\pm\pm\rangle = |\pm\rangle_\tau \otimes |\pm\rangle_\sigma, \tag{6}$$

where $|\pm\rangle_\tau$ and $|\pm\rangle_\sigma$ are two-component states satisfying

$$\begin{aligned}(-\mu\tau_z + \Delta_{4a}k_x\tau_x|\pm\rangle_\sigma &= \pm\sqrt{\mu^2 + (\Delta_{4a}k_x)^2}|\pm\rangle_\sigma \\ (k_x\sigma_y - k_y\sigma_x)|\pm\rangle_\tau &= \pm\sqrt{k_x^2 + k_y^2}|\pm\rangle_\tau.\end{aligned} \tag{7}$$

As σ and τ degrees are independent, the inter-band velocity matrix element between the two bands nearest to the Fermi level can be simply calculated as

$$\begin{aligned}V^a_{+-,-+} &= \frac{1}{\hbar}\langle +-|\partial_a h_0\tau_z|-+\rangle \\ &= \frac{1}{\hbar}\langle +|\tau_z|-\rangle_\tau \langle -|\partial_a h_0|+\rangle_\sigma \\ &= e^{i\phi}v\frac{\Delta_{4a}k_x}{\sqrt{\mu^2 + (\Delta_{4a}k_x)^2}}\frac{k_y}{\sqrt{k_x^2 + k_y^2}}.\end{aligned} \tag{8}$$

for some phase factor ϕ. For $\hbar\omega \sim \Delta_{4a}k_F \ll \mu$, where $k_F = \mu/\hbar v$, the optical conductivity is

$$\begin{aligned}\sigma^{c;c}(\omega) &= \frac{\pi e^2}{2\hbar\omega}\int_{\mathbf{k}}\sum_{n,m} f_{nm}|V^c_{mn}|^2\delta(\omega - \omega_{mn}) \\ &\approx \frac{e^2}{h}\frac{1}{\hbar\omega}\frac{(\Delta_{4a}v_F)^2}{\mu}\frac{\pi}{8}\left(1 - \frac{1}{2}\cos 2\theta_c\right)\end{aligned} \tag{9}$$

in the leading order of ω and Δ_{4a}, where $k^c = |\mathbf{k}|\cos\theta_c$, and θ_c is measured from the x axis.

Supplementary Note 3. An identity on matrix elements of the pairing Hamiltonian

Here we show that $\tau_z H_\Delta$ is a hollow matrix in the BdG energy eigenstate basis, i.e.,

$$\langle n\mathbf{k}|\tau_z H_\Delta(\mathbf{k})|n\mathbf{k}\rangle = 0 \quad \forall n, \tag{10}$$

where $|n\mathbf{k}\rangle$ is an energy eigenstate of the BdG Hamiltonian, and

$$H_\Delta(\mathbf{k}) = \begin{pmatrix} 0 & \Delta(\mathbf{k}) \\ -\Delta^*(-\mathbf{k}) & 0 \end{pmatrix} \tag{11}$$

is the pairing part of the Hamiltonian. It can be simply shown by

$$
\begin{aligned}
\langle m|\tau_z H_\Delta|n\rangle &= \frac{1}{2}\langle m|[\tau_z, H_\Delta]|n\rangle \\
&= \frac{1}{2}\langle m|[\tau_z, H - H_0]|n\rangle \\
&= \frac{1}{2}\langle m|[\tau_z, H]|n\rangle \\
&= \frac{1}{2}\langle m|\tau_z|n\rangle (E_n - E_m) \\
&= 0 \text{ for } n = m,
\end{aligned} \tag{12}
$$

where we use that τ_z and H_Δ anti-commutes in the first line, H_0 is the normal-state Hamiltonian in the BdG form, and τ_z commutes with H_0 in the third line.

This identity can be used to understand why $\sigma^{yy} = 0$ in Fig. 1(d). Let us note that PC changes the M_z eigenvalue λ while S preserves λ for even-M_z pairing such as the Δ_5 pairing. Therefore, a potentially non-zero spectrum-inversion-symmetric excitation is due to the velocity operator between states related by the chiral operation. If we look at the y component,

$$
\begin{aligned}
\langle Sn|V^y|n\rangle &= -\langle n|S\tau_z\rho_x\sigma_x|n\rangle \\
&= \langle n|S\rho_y\sigma_y M_z|n\rangle \\
&= \lambda_n \langle n|S\rho_y\sigma_y|n\rangle \\
&= \frac{\lambda_n}{i\Delta_5}\langle n|\tau_z H_\Delta|n\rangle ,
\end{aligned} \tag{13}
$$

where $M_z = i\tau_z\rho_z\sigma_z$, $S = \tau_x\sigma_y$, and $H_\Delta = -\Delta_5\tau_y\rho_y$. It is zero by the identity we derive above.
